# Study of the Efficiency of a Polycation Using the Diafiltration Technique in the Removal of the Antibiotic Oxytetracycline Used in Aquaculture

**DOI:** 10.3390/membranes13100828

**Published:** 2023-10-10

**Authors:** Daniel A. Palacio, Pablo Oñate, Samir Esquivel, Manuel Meléndrez, Eduardo Pereira, Bernabé L. Rivas

**Affiliations:** 1 Departamento de Polímeros, Facultad de Ciencias Químicas, Universidad de Concepción, Casilla 160-C, Concepción 4070409, Chile; dapalacio@udec.cl (D.A.P.); samiresquivel01@gmail.com (S.E.); 2 Departamento de Ingeniería de Materiales (DIMAT), Facultad de Ingeniería, Universidad de Concepción, Edmundo Larenas 270, Casilla 160-C, Concepción 4070409, Chile; 3 Departamento de Química Analítica e Inorgánica, Facultad de Ciencias Químicas, Universidad de Concepción, Casilla 160-C, Concepción 4070409, Chile; 4 Universidad San Sebastián, sede Concepción, Concepción 4080871, Chile

**Keywords:** ultrafiltration membranes, polycation, removal, oxytetracycline

## Abstract

The presence of antibiotics in aquatic systems in recent years has become a global environmental and public health concern due to the appearance of strains resistant to these antibiotics. Oxytetracycline (OXT) is a high-impact antibiotic used for both human and veterinary consumption, and it is the second most used antibiotic in aquaculture in Chile. Based on the above, this problem is addressed using a linear polymer whose structure is composed of aromatic rings and quaternary ammonium groups, which will help enhance the removal capacity of this antibiotic. To obtain the polycation, a radical polymerization synthesis was carried out using (4-vinylbenzyl)-trimethylammonium chloride as the monomer. The polycation was characterized via Fourier Transform Infrared spectroscopy (FTIR) and Nuclear Magnetic Resonance (NMR). The removal studies were conducted under different experimental conditions such as pH levels (3.0, 5.0, 7.0, 8.0, and 11.0), ionic strength (0.0–0.50 mg L^−1^ of NaCl), polymer dose (0.25–25.5 mg), variation of the antibiotic concentration (1–100 mg L^−1^), and evaluation of the maximum retention capacity, as well as load and discharge studies. The antibiotic retention removal was higher than 80.0%. The antibiotic removal performance is greatly affected by the effect of pH, ionic strength, molar ratio, and/or OXT concentration, as these parameters directly affect the electrostatic interactions between the polymer and the antibiotics. The diafiltration technique was shown to be highly efficient for the removal of OXT, with maximum removal capacities of 1273, 966, and 778 mg OXT g^−1^ polycation. In conclusion, it can be said that coupling water-soluble polymers to the diafiltration technique is an excellent low-cost way to address the problem of antibiotics in aquatic systems.

## 1. Introduction

Water is a crucial resource for life, health, and well-being of all living organisms, as well as for many industries [1,2]. However, in the past few decades, this resource has been significantly impacted by the presence of emerging contaminants (ECs). These contaminants are synthetic and naturally occurring chemicals that are not commonly monitored but have the capacity to enter the environment and cause adverse effects on both the environment and human health [3,4]. One of the pollutants that have raised great concern are antibiotics, which are effective drugs that improve human and animal health [5,6]. These are considered emerging anthropogenic contaminants of major concern because the generated residues present in aqueous systems tend to bioaccumulate and become active compounds that increase the resistance of pathogenic bacteria [7,8]. Therefore, scientists estimate that this drug resistance will cause 10 million deaths worldwide every year until 2050, as they have the capacity to cause allergic hypersensitivity reactions and nephropathy, as well as carcinogenicity, mutagenicity, and toxicity [9,10]. In the case of the salmon industry in Chile, large quantities of antibiotics have been used in relation to their production volumes, with florfenicol and oxytetracycline being the most widely used for the control of *Piscirickettsia salmonis*. Research has shown that recent mutations, such as SLGO94 and SLGO95, of this pathogen present a higher level of resistance to these antibiotics [11,12,13].

Since most antibiotics are soluble in water (especially the antibiotics under study), conventional treatment and separation procedures cannot remove them completely due to the high time spent and the use of harmful organic solvents [14,15]. Currently, many removal and/or elimination techniques such as chemical precipitation, ion exchange, adsorption, coagulation, membrane filtration, and photocatalysis have been implemented to remove emerging contaminants [16,17,18]. Adsorption and membrane separation are among the simplest and most effective techniques for removing organic and inorganic substances. These techniques are characterized by high efficiency, low cost, and ease of operation [19,20]. For example, polymeric adsorbents contain functional groups (ionizable and non-ionizable) like carboxylic acids, amines, amides, and thiols, which allow interaction with organic compounds through non-bonding interactions [21,22]. However, the limitation of this technique is reflected in the possible generation of fouling that may occur during the removal process, which is a major drawback in continuous removal processes in water treatment systems.

The use of membranes by diafiltration is an alternative method and it can be used to transfer a macromolecule or protein from one solvent to another. However, although the use of membranes presents high removal percentages of emerging contaminants, its disadvantage lies in the excess of energy used. Therefore, the need to minimize this problem arises. However, modified methods, such as interfacial polymerization and incorporation of nanoparticles in polymeric membranes like poly(acrylonitrile) (PAN), poly(sulfone amide) (PSA), poly(ether sulfone) (PES), poly(vinylidene fluoride) (PVDF) have now been adopted [20,23,24].

Recent studies have demonstrated the efficacy of polymers in adsorption techniques and separation of substances using filtration membranes. Tessier et al. utilized coupled ultrafiltration–nanofiltration membranes to separate benzylpenicillin (BP). The results showed a 92.4% removal of the antibiotic without the formation of emulsions [25]. Acero et al. investigated the removal of sulfamethoxazole, flumequine, ketorolac, atrazine, isoproturon, 2-hydroxybiphenyl, and diclofenac through micellar-enhanced ultrafiltration (MEUF) with cetylpyridinium chloride (CPC) and cetyltrimethylammonium bromide (CTAB). They found that the cationic surfactant CPC exhibited optimum separation up to 95.0% [26]. Li et al. designed three new porous organic polymers with different m-terphenyl (POP-T) ratios by Friedel–Crafts alkylation. Adsorption results demonstrated that POP-T (1:1.5) exhibited excellent adsorption performance for rhodamine B (RhB), methylene blue (MB), and diclofenac (DCF) [27]. Jiang et al. synthesized nanofibers from L-poly lactic acid (PLLA) and chitosan (CS) coated with graphene oxide (GO) and TiO_2_ to remove methyl orange (MO) and Congo red (CR) dyes. The results showed that the composite nanofiber membranes (PLLA/CS, PLLA/CS-GO, and PLLA/CS-GO/TiO_2_) maintained more than 80.0% of their adsorption capacity after four repeated cycles [28]. Mosavi et al. synthesized a magnetic bionanocomposite through an in-situ copolymerization method using carboxymethyl tragacanth gum in the presence of aniline monomer and γ Fe_2_O_3_ nanoparticles for the removal of amoxicillin from contaminated water. The results showed a maximum adsorption capacity 909.09 mg g^−1^ at pH 7 [29].

Oxytetracycline (OXT) is an antibiotic from the tetracycline type and chemically belongs to the polyketide family. It has the ability to exhibit antibiotic activities against infections caused by Gram (+) and Gram (−) microorganisms [4,30]. It is commonly used in aquaculture, but due to its solubility in water it shows poor absorption and 30–90% of the parent compound is excreted [31,32]. Research has even confirmed the presence of oxytetracycline in wastewater and surface water with concentrations ranging from 0.0003 to 340 ng L^−1^ [9,33]. The objective of this research is to study the retention effect of oxytetracycline (highly used in aquaculture) from poly[(4-vinylbenzyl)-trimethylammonium chloride] polycation; coupled to the diafiltration technique as a previous step to degradation processes; to reduce the concentrations of this antibiotic in water and decrease the appearance of new resistant bacterial strains due to the presence of these contaminants in aqueous systems.

## 2. Materials and Methods

### 2.1. Materials

The reagents used for antibiotic removal were oxytetracycline hydrochloride (OXT, 96.5% purity for HPLC) for weekly standard preparation. Hydrochloric acid standards 0.1 mol L^−1^ (HCl) and sodium hydroxide 0.1 mol L^−1^ (NaOH) were used to adjust pH of the solutions under study. Sodium chloride (NaCl, 99.6%) was used for the evaluation of ionic strength. The monomer used to obtain the homopolymer electrolyte (HPE) was [(4-vinylbenzyl) trimethyl ammonium chloride], and ammonium persulfate (APS 98%) used as radical initiator. All the solutions used in this investigation were prepared with deionized water type I and all the reagents were purchased from Sigma Aldrich; Darmstadt, Germany.

### 2.2. Synthesis of Polycation—Homopolymer Polyelectrolyte (HPE)

The polyelectrolyte homopolymer poly[4-vinylbenzyl) trimethylammonium chloride] was synthesized via free radical polymerization. A quantity of 0.2 mol of monomer was dissolved in 40 mL of deionized water. Subsequently, 1.0 mol % ammonium persulfate (APS 98%) as initiator was added to the amount of dissolved monomer and degassed for 10 min under nitrogen atmosphere. Polymerization was carried out for 24 h at 80 ± 1 °C under nitrogen atmosphere. For its purification, the obtained product was dissolved in 2000 mL of deionized water and ultrafiltered with regenerated cellulose membranes with exclusion weight of 100 kDa (76 mm diameter, Millipore-Ultracel, Rahway, NJ, USA) and working pressure between 55.0 and 65.0 psi. It was then lyophilized and subsequently characterized [34].

### 2.3. Characterization

*Fourier transform infrared spectroscopy (FTIR):* the polyelectrolyte, antibiotic, and polymer-antibiotic were evaluated by FTIR spectroscopy. To determine the functional groups, a Nicolet spectrometer equipped with a DTGS-KBr detector (Thermo Fisher Scientific, Waltham, MA, USA), was used and analyzed in the range of 400–4000 cm^−1^.

*Nuclear Magnetic Resonance (^1^H-NMR):* the homopolymer, antibiotic, and polymer-antibiotic structure was analyzed using 600 µL of deuterated water (D_2_O) to dissolve 20 mg of each sample separately. The analyses were performed using a Bruker Advance 400 MHz instrument- (Bruker, Billerica, MA, USA).

*Scanning electron microscopy (SEM):* it was used to study the surface characteristics of the homopolymer and polymer-antibiotic by scanning electron microscopy (SEM) using a JEOLSEM-PROBE CAMECA model SU-30, equipped with an EDS detector-Tokyo, Japan.

### 2.4. OXT Removal Using Diafiltration Technique

**Figure 1:**
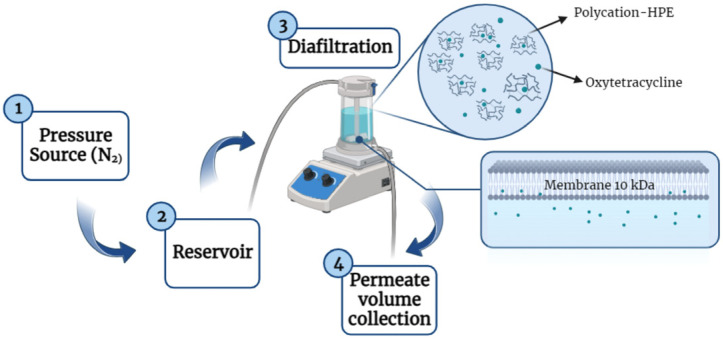
Schematic representation of the OXT removal process using the diafiltration technique.

To evaluate the removal of oxytetracycline using poly(4-vinylbenzyl) trimethylammonium chloride) and determine the ideal conditions to improve its performance, the diafiltration technique was used (Figure 1). The effect of pH, ionic strength, increase in polymer, and antibiotic concentration using the washing method in an aqueous medium was studied. The maximum retention capacity was evaluated using the enrichment method. The antibiotic concentration was determined using a (Thermo Scientific Evolution One Pluss spectrophotometer-(Thermo Fisher Scientific, Waltham, MA, USA)) with 1 cm optical path quartz cuvettes. The system used in this technique consists of a 50 mL ultrafiltration cell with a magnetic stirrer (Amicon 8050, Millipore, Merck, Rahway, NJ, USA), which contains a 10 kDa exclusion size regenerated cellulose membrane (Ultracel PLGC, Millipore, Merck, Rahway, NJ, USA), a 1000 mL reservoir (Amicon RC800, Millipore, Merck, Rahway, NJ, USA), Amicon selector (Millipore, Merck, Rahway, NJ, USA), and a 99.995% purity nitrogen gas pressure source. Two methods were used for this purpose: the washing method and the enrichment method [35,36].

#### 2.4.1. Washing Method

The washing method was used to evaluate the best conditions for pH, ionic strength, molar ratio (Antibiotic: polycation-HPE), and antibiotic concentration. The same procedure was followed for each parameter evaluated, maintaining a constant cell volume of 20 mL and collecting fractions of the permeate with the same volume until completing 10 test tubes per analysis [37]. From a standard of 1000 mg L^−1^ antibiotic, solutions of 30 mg L^−1^ were prepared in deionized water with molar ratios of 1:20 of antibiotic and polymer. The pH was adjusted using 0.1 mol L^−1^ NaOH or HCl, and experiments were conducted separately at pH values of 3.0, 5.0, 7.0, 9.0, and 11.0. The effect of ionic strength was evaluated by maintaining an OXT concentration of 30 mg L^−1^, a molar ratio of 1:20 (OXT: polycation-HPE) at the pH value that showed the best performance. Separate experiments were performed by varying the NaCl concentration at 0.025, 0.050, 0.100, 0.25, and 0.500 mg L^−1^.

Then, we proceeded to study the increase in the molar ratio while maintaining the same concentration of the antibiotic with the optimal parameters of pH and ionic strength. In separate experiments, we evaluated molar ratios of 1:1, 1:5, 1:20, 1:40, and 1:100 (OXT: polycation-HPE). Once we determined the pH, ionic strength, and molar ratio parameters that yielded the best performance in OXT retention, we proceeded to evaluate the increase in antibiotic concentration. We evaluated concentrations of 1.0, 5.0, 30.0, 40.0, 80.0, and 100.0 mg L^−1^. The results obtained were plotted as F (Vp/Vc) Vs Rpol (%), where F is given by the filtration factor which is the result between the permeate volume (Vp) and the cell volume (Vc). While, Rpol (%) is the retention of the polymer expressed in percentages.

#### 2.4.2. Enrichment Method

The enrichment method was used to determine the maximum retention capacity (MRC) of the polymer for OXT [37]. Using the conditions of pH, ionic strength, molar ratio, and antibiotic concentration that yielded the highest results were used for the experiment. A constant cell volume of 20 mL was maintained under optimal conditions, and it was enriched with a solution of the same antibiotic concentration from the reservoir. Filtrate volumes of 20 mL were taken until the maximum polymer retention capacity was achieved. Additionally, a washing method with pH water was necessary, where the lowest percentage of removal was observed. This washing method was carried out after each enrichment to extract the antibiotic from the solution. The same procedure was repeated for three cycles.

### 2.5. OXT Removal Using Diafiltration Technique

The data are presented as the mean ± standard deviation. One analysis of variance (ANOVA) with Tukey’s test was used to analyze the significant differences between samples. For all tests, the level of significance was set to * *p* < 0.05, ** *p* < 0.01, *** *p* < 0.001, and **** *p* < 0.0001.

## 3. Results and Discussion

### 3.1. Characterization of HPE

**Figure 2:**
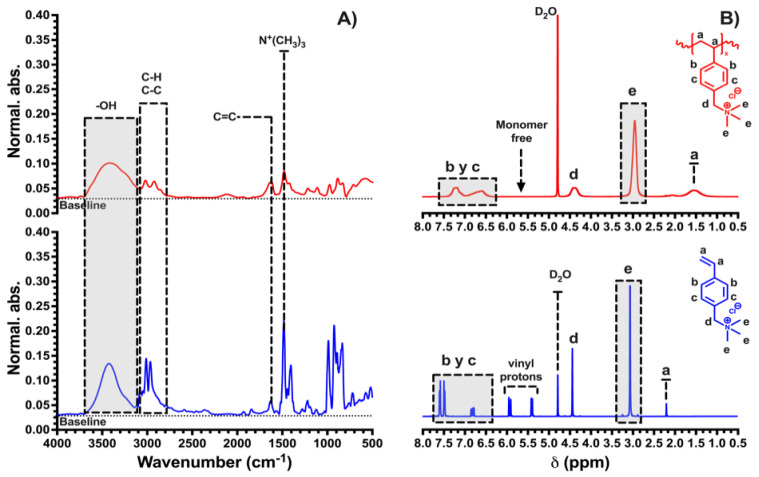
Spectroscopic characterization using, (**A**) ^1^H-NMR, and (**B**) FTIR-ATR. Spectra in blue correspond to the monomer and spectra in red correspond to the polycation-HPE.

In Figure 2A, the FTIR spectra are presented to confirm the presence of the functional groups in the HPE. It shows characteristic absorption bands at 3020 cm^−1^ corresponding to C-H bond stretching, 2925 cm^−1^ C-C bond stretching, a strong absorption band at 1482 cm^−1^ characteristic of the quaternary amino group (-N^+^(CH_3_)_3_), and from 1623–1425 cm^−1^ attributed to the C=C bonds of the aromatic ring. Additionally, a strong signal at 3416 cm^−1^ is observed, which is characteristic of the OH groups and is attributed to the hydration of the polymer.

Figure 2B shows the ^1^H-NMR spectra for both the homopolymer and its monomer. Characteristic signals between 2.5 and 3.0 ppm are attributed to the protons present in the (-N^+^(CH_3_)_3_) group [38]. There is also a small peak between 4.0 and 4.5 ppm corresponding to the protons of the methyl group linked to the amino group. Additionally, two displacements between 6.5 and 7.5 ppm correspond to the protons of the aromatic group. The absence of vinyl proton signals, which typically appear between 5.5 and 6.5 ppm, indicates the absence of residual monomers. Based on the results presented by the ^1^H-NMR and FTIR spectra, the structure of the polycation-HPE is confirmed.

### 3.2. Removal Studies

**Figure 3:**
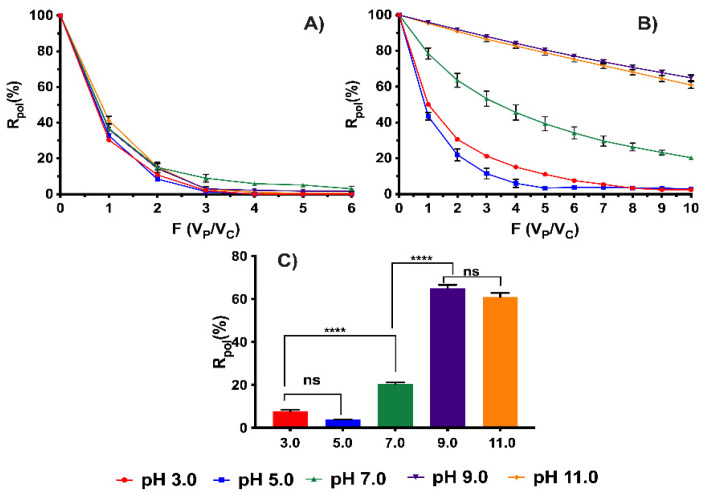
Retention studies as a function of pH at 30.0 mg L^−1^ of OXT: (**A**) Blank experiments (without polymers), (**B**) Retention efficiency in the presence of polycation-HPE, (**C**) Comparative study of the maximum retention efficiency at a volume of 200 mL diafiltrate.

The effect of pH plays an important role in the study of OXT removal due to the speciation of the antibiotic’s functional groups. Depending on the aqueous medium, OXT can be found at different pH due to its pKa, specifically at pKa = 3.2, 7.4, and 8.9 [39]. However, the speciation of oxytetracycline will depend on the water systems in which it is found; generally environmental cultivation systems are between a pH range of 6.5 and 8.5. The Figure 3 shows the results obtained from the pH variation studies (3.0, 5.0, 7.0, 9.0, and 11.0) and using a concentration of OXT at 30 mg L^−1^ at a molar ratio of 1:20 (OXT: polycation-HPE). In Figure 3A, the results of the studies in the absence of the polymer are shown, demonstrating that the membrane does not have a significant effect on the retention of the antibiotic and does not affect the obtained results. It should be noted that the retention equilibrium is achieved approximately 1 h after the end of each experiment.

In Figure 3B, a clear tendency to decrease the percentages of polycation-HPE retention can be observed as the pH decreases. This is mainly due to the effect of electrostatic repulsions, as the OXT is in its cationic state at pH close to 3.27. This pH value is characteristic of the dissociation of the dimethylamine present in the structure [40], which is repelled by the active sites of the polymer (quaternary ammonium groups). On the other hand, as the pH becomes more basic, the highest percentages of removal occur due to the presence of negative charges resulting from the speciation of the dicetone and tricarbonyl groups with the functional groups of the polymer [41].

Figure 3C presents the results of the maximum retention capacity after a wash volume of 200 mL. It can be observed that at pHs 3.0 and 5.0, there are no significant differences in the removal percentages due to the charge repulsion. However, there are no significant differences between pH 7.0 and pH 3.0 or pH 5.0. This can be explained by the fact that at pH 7.0, the OXT molecule is in its zwitterion state [42]. This state causes certain electrostatic interactions with the polyelectrolyte due to the counterion effect present at one end of the molecule, as well as repulsions because of the positive charges at the other end. Meanwhile, at pH 9–11, there are no significant differences in the removal percentages, but there are significant differences observed compared to pH 7.0. For example, a removal percentage of 64.94% is obtained at pH 9.0. These results are mainly influenced by the high electrostatic interactions. However, at pH 11.0, the removal effect is diminished and is not significant. At pH 9.0, the removal effect is diminished and not significant. The slight decrease in the removal percentages at this pH may be influenced by the fact that OXT behaves as a di-anionic molecule, which causes effects of competition of the same molecule for the active sites of the polycation-HPE, thus decreasing the retention efficiency. Similar results were obtained by Harja et al., who studied the adsorption process of oxytetracycline from hydroxyapatite nanopowders. The adsorption results showed that at pH 8 they obtained high oxytetracycline removal rates of 97.58% and 89.95% for uncalcined and calcined nanohydroxyapatites, respectively [43]. On the other hand, Lye et al. evaluated three types of natural zeolites (NZ01, NZ02, and NZ03) for the removal of tetracycline (TC) and oxytetracycline (OXT). The results showed a higher adsorption capacity for sample N202 at pH 7 and 8 with percentages of 62.5 and 76.3 μmol g^−1^ for TC and OXT, respectively [44].

**Figure 4:**
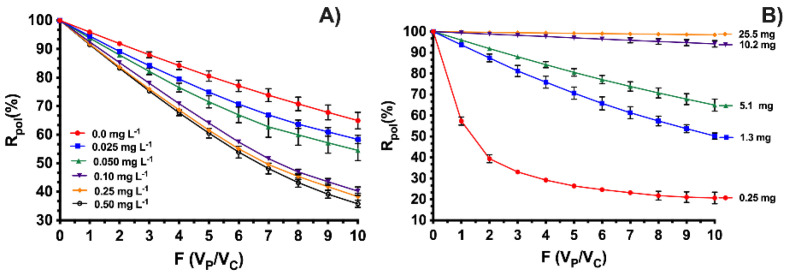
Retention profiles at pH 9.0 and 30.0 mg L^−1^ of OXT, as a function of (**A**) NaCl concentration variation and (**B**) polycation dose.

For the studies of the effect of ionic strength, a concentration of 30 mg L^−1^ and pH 9.0 was used. The retention profiles in Figure 4A show the negative effect of increasing ionic strength on the removal of OXT. Similar results were found by Liang et al., who obtained a montmorillonite (MMT)–biochar (MBC) composite and a magnetic MMT–biochar composite (MMBC), for the adsorption of oxytetracycline. They found that with increasing Ca^2+^ concentrations, the adsorption capacities were slightly reduced for all adsorbents at the same OXT concentration, due to the competitive effect for adsorption sites between the Ca^2+^ cation and OXT [45]. On the other hand, Zhang et al. prepared a magnetic Fe_3_O_4_-graphene nanocomposite by in situ precipitation method for the removal of oxytetracycline (OXT) and tetracycline (TC) from aqueous solution. The results indicated that the addition of NaCl had an obvious effect on the adsorption of OXT and TC. They observed that the adsorption capacity decreases with the addition of NaCl, due to the electronic sensing effect of surface charge sites caused by the increase in the Na^+^ weakened cation-π bond [46]. This trend can be explained by the competition presented by chloride ions towards anionic OXT for the active sites of the polyelectrolyte, in addition to the interactions of sodium ions with the negative charges of the antibiotic itself. Another reason for the significant decrease in the retention of OXT is explained by the behavior of the polymer due to the increase in ionic strength in the solution, causing agglomeration effects and preventing the interaction of OXT and polycation-HPE. At low ionic strengths, the polycation-HPE chains can be presented in an extended form, which favors the interaction between the active sites of the HPE and OXT. Additionally, it can be explained that, according to Solis et al. (2001), the presence of a high concentration of counterions produces ionic bridges between the charged groups of the polyelectrolyte chain, causing it to remain extended but preventing interactions with the antibiotic molecules. Furthermore, the formation of complexes between the antibiotic chain and the polymer can occur, causing large amounts of salt that break the necessary interactions for efficient removal [47].

By varying the dose of polycation-HPE in Figure 4B, an increase in the retention of the polymer can be observed, improving the retention efficiency and reaching percentages higher than 95.0% using doses of 25.5 mg of polymer and an antibiotic concentration of 30 mg L^−1^. This is influenced by the increase in the number of molecules of polycation-HPE in the solution, which considerably increases the presence of active sites that can interact with the OXT without creating competition for them. This shows that the number of active sites has a direct relationship with the removal efficiency, going from 64.94% with 5.1 mg of HPE to 98.50% with the use of 25.5 mg of HPE. Recent studies support this claim. For example, Li et al. prepared coconut shell biochar (BC), stripped biochar (HBC), and nanovalent iron-loaded biochar (nZVI-HBC) to remove oxytetracycline. The results showed that the adsorption rate of OXT increases linearly with the increasing addition of BC, HBC, and nZVI-HBC, yielding a removal rate of 79.92% for BC and 91.19% for nZVI-HBC, confirming that there are more active sites with increasing adsorbent material [48]. Hadki et al. obtained graphene oxide (GO) and boron-doped reduced graphene oxide (B-rGO) adsorbent materials through an oxidation/exfoliation process using the modified Hummers method. Additionally, they obtained B-rGO through a thermal annealing approach for the removal of oxytetracycline. The data showed that the removal efficiency of OXT significantly improved from 61.0% and 69.0% to reach 87.0% and 97.0%, respectively, as the dose increased from 0.1 to 0.3 g L^−1^ for GO and B-rGO [49].

**Figure 5:**
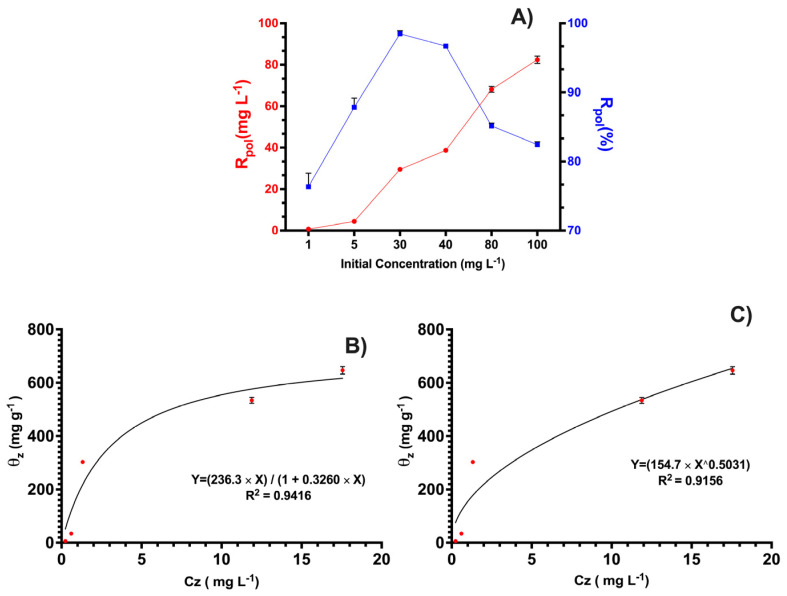
(**A**) Effect of OXT removal as a function of concentration, (**B**) Langmuir, and (**C**) Freundlich adsorption isotherms.

The retention of the polymer when varying the initial concentration of OXT is an important factor in determining the effective retention capacity of the polycation-HPE. Figure 5A shows the retention of the polymer when varying the concentration of OXT as a function of mg L^−1^ of retained concentration and percentage. It can be observed that as the initial concentration of OXT increases, the retention capacity of the polycation also increases. However, when considering the percentages, it reaches a maximum retention rate of approximately 98.0% at a concentration of 30 mg L^−1^ and begins to decrease as the initial concentration of OXT increases. This effect may be related to the limited number of active sites or functional groups that can interact with OXT, indicating that at certain antibiotic concentrations, the polymer reaches saturation. Additionally, the ability of the polymer to increase retention at equilibrium with increasing OXT concentration is appreciated, thanks to concentration gradient effects that act as driving forces for the transfer of antibiotics between the antibiotic solution and the polycation [50,51].

Similarly, non-linear adsorption isotherm models were applied to the equilibrium data obtained for Langmuir (Equation (1)) and Freundlich (Equation (2)) [52]. These models are mainly performed in solid–liquid study systems. However, in studies carried out, these models can be used to determine the interaction between the antibiotic and the polymer surface [53,54].

(1) $$\theta_{z}=\frac{Q_{zmax}K_{L}C_{z}}{1+K_{L}C_{z}}$$

(2) $$\theta_{z}=K_{F}C_{z}^{\frac{1}{n}}$$

where, $\theta_{z}$ (mg g^−1^) corresponds to the amount of OXT bound to the polycation-HPE per monomer unit, $C_{z}$ (mg L^−1^) is the concentration of the OXT not retained in the polycation-HPE, *K_F_* is the Freundlich constant, n expresses the value of the heterogeneity retention and intensity factor, and *K_L_* is the Langmuir constant. R^2^ is the correlation coefficient, which allows us to evaluate the fit of the isothermal models.

From the results obtained in Figure 5A,B, it can be said that the Langmuir model explains the interactions of polycation-HPE with OXT most effectively, as it has the highest correlation coefficient of 0.94. This may be due to the Langmuir model considering a fixed number of active sites that promote interactions and assuming equal energy for these interactions. Additionally, the model assumes reversibility of the interactions, with a preference for occupation of the active sites and no interaction between OXT molecules in this case [55,56]. Similar results were found by Oyarce et al. They studied the removal effect of methyl orange and methylene blue dye using the polymer-enhanced diafiltration technique, reporting that their data fitted Langmuir isotherms [54]. Similarly, the Langmuir model reveals that the polymer can have approximate maximum adsorption capacity of 724.9 g g^−1^ in discontinuous systems. However, its maximum saturation capacity can increase in discontinuous studies, as observed in the charge and discharge studies.

### 3.3. Charge–Discharge

The charge and discharge studies are important studies that help us determine the capacity of materials to be used in different cycles. It is also important to know the maximum saturation capacity of the material in order to know to what extent the material can be used to support a certain content of analyte to be removed. In the charge–discharge processes, the washing method and the enrichment method were used in parallel. By means of the enrichment method, the maximum retention capacity (MRC) of the polycation-HPE could be calculated according to the following Equation (3):

(3) $$Polycation-HPE_{MRC}=\frac{\left[OXT_{initial}\right]\times V_{saturation}}{Policatyon-HPE_{mass}}$$

where $\left[OXT_{initial}\right]$ is the initial OXT concentration in mg L^−1^ used in the reservoir, $policatyon-HPE_{mass}$ is the mass of policatyon−HPE used in the retention experiments, and $V_{saturation}$ is the total volume expended to reach saturation of the FSPs. To know the charge effect, an antibiotic solution of 30 mg L^−1^, a polymer dose of 25.5 mg, pH 9.0 and null ionic strength were used, obtaining different saturation volumes in each charge cycle of 1200 mL, 980 mL, and 800 mL for each respective cycle. Being this, representative to the maximum retention capacity obtained of 1273 mg g^−1^, 966 mg g^−1^, and 778 mg g^−1^. It demonstrated the high saturation capacity presented by this polymer for its three consecutive loads, after each desorption process. These results are shown also to be highly efficient and competitive when compared with reported studies of materials for the removal of OXT in aqueous solutions (see Table 1).

**Figure 6:**
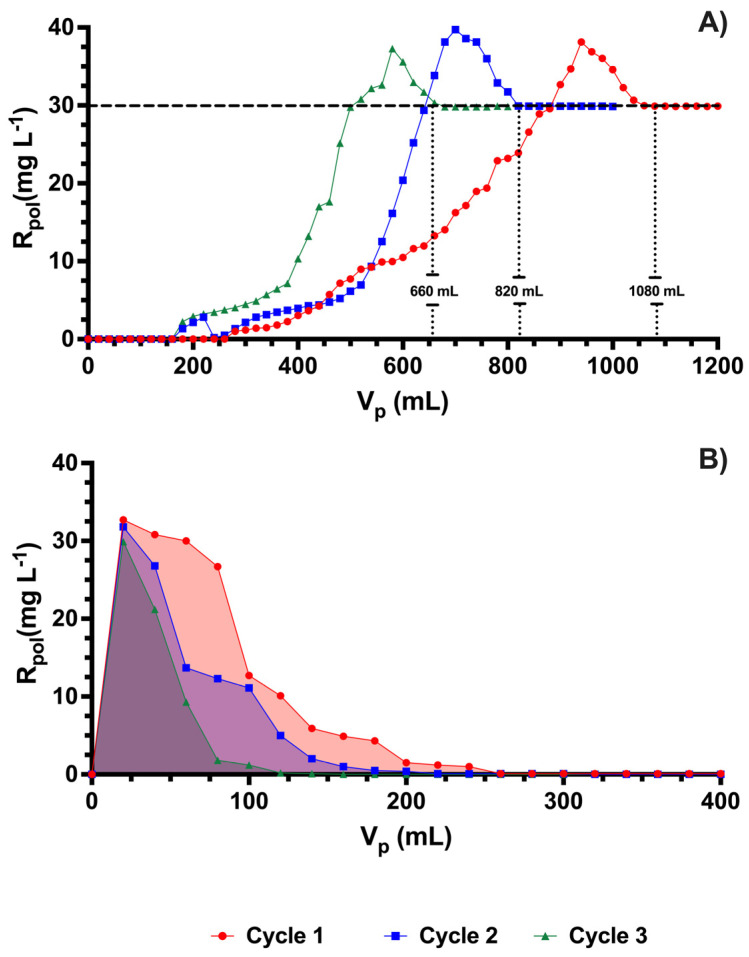
Charge–discharge cycles, (**A**) and (**B**), respectively.

Figure 6B shows the behavior of discharge or desorption of OTX which can be necessary to know the useful life of the polycation-HPE. For this case, water at pH 3.0 was used, where the lowest interactions between the polymer and OTX occurred. The three desorption processes using water at pH 3.0 proved to be efficient because in acidic conditions the antibiotic is in its cationic state being repelled by the active sites of the polymer which are positively charged, thus increasing the discharge processes. On the other hand, the smaller the area of the discharge curve, the faster the discharge curve becomes because the polymer is partially saturated. The larger the area of the curve, the more difficult it is to discharge the antibiotic because in this process the active sites of the polycation-HPE start to become more occupied. In all cases, it can be said that the use of acidic water for this purpose is more convenient than increasing the ionic strength of the system since this presents a greater expense of volume and time, which are important factors in reducing the cost of this process if it were to be implemented on a large scale. This is since when comparing the removal obtained at pH 3.0, which was 0.0% in the 50 mL of permeate, the experiment of increasing the ionic strength with the lowest removal result is 35.8% in the 200 mL of permeate.

**Figure 7:**
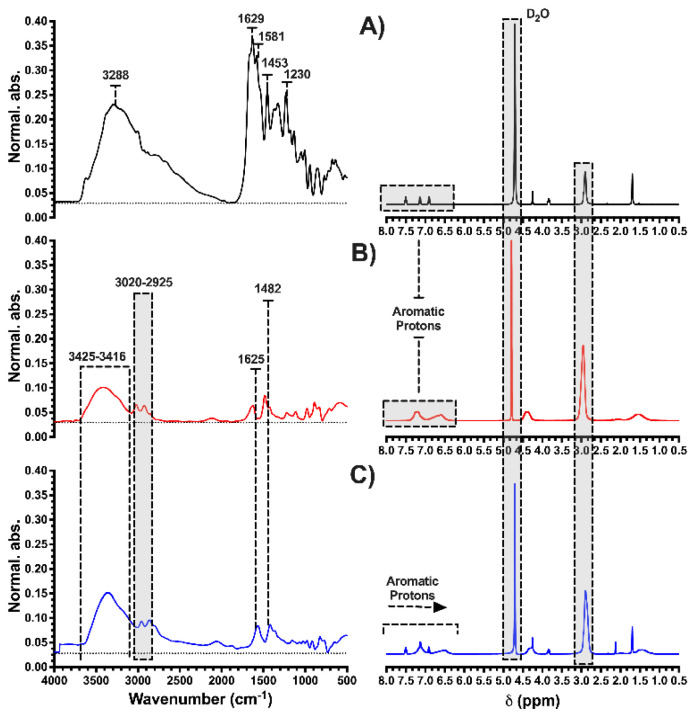
Results of the characterization of the polymer after the removal process at pH 9.0 by FTIR-ATR and ^1^H-NMR (D_2_O, TMS, 400 MHz, and adjusting at pH 9.0 with 0.01 M NaOD). (**A**) OXT, (**B**) polycation-HPE, and (**C**) polycation-OXT.

After studying the ideal conditions for OXT removal, ^1^H-RMN, FTIR, and SEM studies were carried out to predict the mechanisms of interactions between the antibiotic and the polymer. For this purpose, characterization of the product of the contact between the lyophilized polymer-antibiotic, resulting from the experiment with the highest removal performance, was performed. Figure 7 shows the spectra of the polycation-HPE, the antibiotic, and the product resulting from the contact studies between the OXT and the polycation HPE. In the FTIR spectra, the characteristic absorption bands of each component are confirmed. However, the lower intensity is observed for the polycation-OXT spectra with respect to the polycation and lower than that of the OXT, which is influenced by the presence of OXT in the polycation chain, as well as slight shifts corresponding to the (-N^+^(CH_3_)_3_) group. In addition, a clear decrease and slight shift in the OXT bands produced by the C=C bonds of the aromatic ring (produced by a possible stacking effect between the rings in both molecules) is observed. The NMR spectra show slight shifts in the signals corresponding to the protons of the aromatic rings of the polycation, as well as the signals of the aromatic protons of the OXT, which could be explained by the stacking effect between the rings in both molecules [65]. Finally, it demonstrates the presence and interaction of the antibiotic with the polymer in the solution after removal.

**Figure 8:**
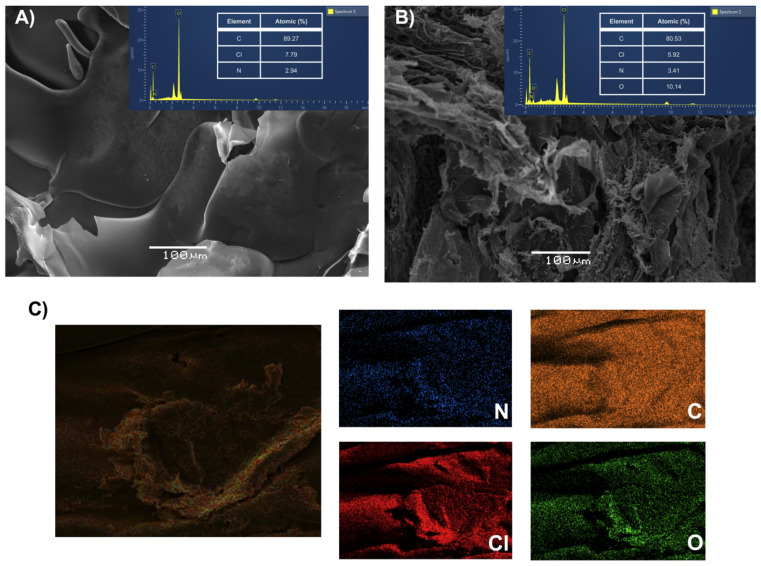
SEM images and EDS spectra of the polycation-HPE, (**A**) before the removal process, (**B**) after the removal process, and (**C**) elemental mapping after the removal process.

Also, SEM images were obtained to know the morphology before and after the retention process of the polycation-HPE. In Figure 8A,B, it can be evidenced that the morphology of the material changes after the removal process, this may be mainly due to the number of molecules that are interacting with the polymer. Moreover, by means of EDS analysis it could be evidenced the increase in nitrogen content after the retention process this due to the nitrogen in the OXT molecule. A decrease in the presence of the counterion Cl^−1^ of the quaternary ammonium group coming from the polymer, after the retention process due to the ion exchange effects between the positive charges of the quaternary ammonium group and the OXT in the anionic state at pH 9.0. As well as the presence of oxygen elements, associated to the OXT molecule, because the polycation-HPE is absent of this element. Likewise, to complete the analysis, Figure 8C shows the images of the mapping of the polycation-HPE after the retention process, confirming the intensity of the elements and the presence of a new element, which provided conclusive evidence of the successful retention of OXT in the polycation-HPE.

## 4. Conclusions

In this research, a cationic poly[(4-vinylbenzyl)-trimethylammonium chloride] polyelectrolyte homopolymer was synthesized from [(4-vinylbenzyl) trimethyl ammonium chloride] to be coupled with ultrafiltration membranes and used for the removal of oxytetracycline in aqueous solution, which is a commonly used antibiotic in the salmon industry. The polycation was analyzed by FTIR and NMR, confirming the presence of functional groups and the absence of vinyl proton from the monomer. In pH studies, OXT removal rates of up to 80.0% were achieved at pH 9.0 with polymer doses of 25.5 mg. In ionic strength studies, a decrease in removal efficiency was observed as the salt concentration in the medium increased, indicating competition between chloride ions and the anionic OXT for the active sites of the polycation. It was also observed that increasing the polymer concentration resulted in an increase in the retention capacity in terms of mg L^−1^, with the polymer retaining approximately 94.0 mg L^−1^ from an initial concentration of 100 mg L^−1^. Regarding the capacity for use and deboning cycles, the polymer demonstrated maximum retention capacities of 1273 mg g^−1^, 966 mg g^−1^, and 778 mg g^−1^ for three respective loading cycles. Based on the results obtained, it can be concluded that this polymer, when coupled with diafiltration techniques, is an efficient and cost-effective method for reducing the environmental impacts caused by oxytetracycline in water.

## Figures and Tables

**Table 1:** Comparison of the MRC for OXT retention on various materials reported in the literature.

Material	Retention Capacity of OXT (mg g^−1^)	Removal Efficiency (%)	Reference
Activated Carbon	522.6	99.9	[57]
Halloysite nanoclay	52.4	68	[58]
Iron oxide (Fe_3_O_4_) modified with polyethylenimine (PEI) and coated with polyoxyethylene sorbitan triolate (Tween 85).	277.8	98	[59]
Silicone-based polydimethylsiloxane (PDMS)	8.38	99.58	[60]
Fe-MetalOrganic Framework/Biopolymer-Clay Hydrogel.	26.14	94	[61]
Alumina modified with the surfactant sodium dodecyl sulfate (SDS) (SMA).	143	97	[62]
Zn/Fe double layer hydroxide hydroxide (LDH).	90.3	77.23	[63]
Cu/Al-doped nitrogen-containing carbon microspheres prepared from chitosan.	1727.65	92.25	[64]
Polycation-HPE	1273.0	80	Current study

## Data Availability

Not applicable.
